# Efficient and Selective Removal of Palladium from Simulated High-Level Liquid Waste Using a Silica-Based Adsorbent NTAamide(C8)/SiO_2_-P

**DOI:** 10.3390/nano14060544

**Published:** 2024-03-20

**Authors:** Jiaxuan Shi, Junli Wang, Wentao Wang, Xuan Wu, Hui Wang, Jianwei Li

**Affiliations:** 1 State Key Laboratory of Chemical Resource Engineering, College of Chemical Engineering, Beijing University of Chemical Technology, Beijing 100029, China; 2 Department of Radiochemistry, China Institute of Atomic Energy, Beijing 102413, China

**Keywords:** palladium(II), adsorption, high-level liquid waste, NTAamide(C8)/SiO_2_-P adsorbent

## Abstract

In order to realize the effective separation of palladium from high-level liquid waste (HLLW), a ligand-supported adsorbent (NTAamide(C8)/SiO_2_-P) was prepared by the impregnation method in a vacuum. The SiO_2_-P carrier was synthesized by in situ polymerization of divinylbenzene and styrene monomers on a macroporous silica skeleton. The NTAamide(C8)/SiO_2_-P adsorbent was fabricated by impregnating an NTAamide(C8) ligand into the pore of a SiO_2_-P carrier under a vacuum condition. The adsorption performance of NTAamide(C8)/SiO_2_-P in nitric acid medium has been systematically studied. In a solution of 0.2 M HNO_3_, the distribution coefficient of Pd on NTAamide(C8)/SiO_2_-P was 1848 mL/g with an adsorption percentage of 90.24%. With the concentration of nitric acid increasing, the adsorption capacity of NTAamide(C8)/SiO_2_-P decreases. Compared to the other 10 potential interfering ions in fission products, NTAamide(C8)/SiO_2_-P exhibited excellent adsorption selectivity for Pd(II). The separation factor (SF_Pd/other metals_ > 77.8) is significantly higher than that of similar materials. The interference of NaNO_3_ had a negligible effect on the adsorption performance of NTAamide(C8)/SiO_2_-P, which maintained above 90%. The adsorption kinetics of Pd(II) adsorption on NTAamide(C8)/SiO_2_-P fits well with the pseudo-second order model. The Sips model is more suitable than the Langmuir and Freundlich model for describing the adsorption behavior. Thermodynamic analysis showed that the adsorption of Pd(II) on NTAamide(C8)/SiO_2_-P was a spontaneous, endothermic, and rapid process. NTAamide(C8)/SiO_2_-P also demonstrated good reusability and economic feasibility.

## 1. Introduction

Nuclear energy, as a clean and reliable energy source, is favored by many countries due to its unique advantages. High-level liquid waste (HLLW) generated from fuel treatment contains more than 40 elements, such as Ru, Rh, Pd, Tb, Tc, Cs, Sr, Mo, I, etc. [1,2] Palladium is a platinum-group metal with a silver luster. The palladium in the HLLW includes stable isotopes ^104^Pd (17 wt.%), ^105^Pd (29 wt.%), ^106^Pd (21 wt.%), ^108^ Pd (12 wt.%), and ^110^Pd (4 wt.%), and radioactive isotope ^107^Pd (17 wt.%). The half-life of ^107^Pd is 6.5 × 10^6^ years. Compared with other elements in HLLW (such as Rh and Ru), the radioactivity is very weak and considered safe to recover [3,4,5]. During the subsequent treatment of HLLW through vitrification, palladium tends to deposit at the bottom of the furnace because of its high melting point and low solubility in the high-level radioactive nuclear waste glass, which greatly reduces the stability of the glass body and increases the difficulty of vitrification. To enhance the vitrification efficiency, palladium needs to be extracted from HLLW in advance. On the other hand, due to the excellent catalytic properties, thermal stability, corrosion resistance, and other characteristics of palladium, it is an important strategic material with economic and industrial value [6,7] and has been widely used in catalysis [8], actor manufacturing [9], aerospace [10], automobile [11], biomedicine [12], and other aspects. Palladium, like other platinum group metals, has a scarce reserve, and its supply always requires secondary resources [13]. Comprehensive treatment of secondary resources and efficient recycling of Pd are of great significance to increase the supply of Pd and promote the sustainable development of multiple industries [14,15,16,17]. Therefore, recovering palladium from HLLW not only facilitates the subsequent treatment of HLLW through vitrification, but also makes use of the secondary resources to generate Pd.

**Figure 1:**
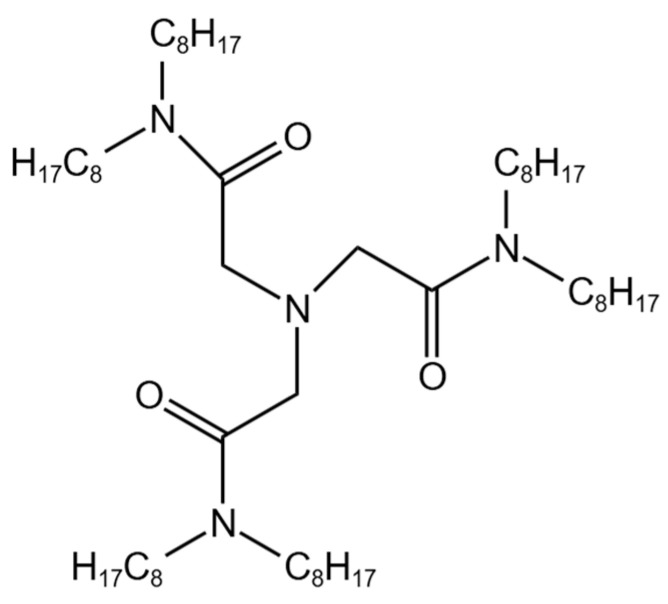
Structure of NTAamide(C8).

Solvent extraction is a widely used method for recovering palladium from HLLW [1,2,18,19]. It is well known that Pd(II) belongs to a soft Lewis acid, and can easily complex with the ligand containing a soft-donor N or S atom [20]. *N,N,N′,N′,N″,N″*-hexaoctyl-nitrilotriacetamide (NTAamide(C8)) has received increasing attention, as a new soft non-heterocyclic N-donor extractant. The structure of NTAamide(C8) is shown in Figure 1. Sasaki et al. [21] used NTAamide(C8) to recover Pd(II) through extraction electrodeposition, and the extraction rate could reach above 91% in all cycles. Wang et al. [22] studied the extraction ability of NTAamide(n-Oct) for Pd(II) and confirmed its good selectivity, extraction efficiency, and loading capacity. Sasaki et al. [23] studied the extraction behavior of NTAamide(C8) for 70 metals and found that the extraction rate for Pd(II) was the highest, indicating that NTAamide(C8) had good selectivity for Pd(II) and could be used to separate Pd(II) from multi-element mixed solutions, such as HLLW.

Despite the high recovery efficiency and the potential to scale up, solvent extraction methods suffer from emulsification and prominent three-phase problems. The extraction process also consumes a large volume of solvents and generates toxic organic waste. In addition, solvent extraction has high requirements for subsequent treatment and economic investment. Due to these drawbacks, some alternative separation methods have been developed [24,25,26,27,28]. The chemical adsorption method [25,28,29] is widely used due to its high efficiency and low cost, especially for recovery metals at low concentrations. At present, the adsorbents mainly include inorganic material adsorbents, nanomaterial adsorbents, ion exchange resin adsorbents, and biomaterial adsorbents. Awual et al. [30] prepared a highly selective and efficient adsorbent by immobilizing the synthesized *N,N*-bis(salicylidene)1,2-bis(2-aminophenylthio)ethane ligand onto mesoporous silica monoliths, and its maximum sorption capacity for Pd(II) ions was 163.13 mg/g. Mincke et al. [31] synthesized three chitosan derivatives and used them to recover Pd(II) and Pt(IV) from acidic solutions. These absorbents showed high adsorption rates, reaching equilibrium within 30 min. Their maximum adsorption capacities for Pd(II) were 262.6 mg/g, 154.7 mg/g, and 340.3 mg/g, respectively. However, due to the coexistence of platinum group metals in HLLW and their similar chemical properties, adsorbents tend to adsorb them simultaneously, making it impossible to selectively adsorb Pd. This needs further in-depth study.

SiO_2_-P is a composite carrier composed of porous silica and organic copolymers. The adsorbents based on SiO_2_-P demonstrate a high adsorption rate, good reusability, and environmental friendliness. Yu et al. [32] prepared a macroporous silica polymer (TRPO/SiO_2_-P) to recover scandium from sulfuric acid solutions. The adsorption was selective, reaching an equilibrium within 120 min with a saturated adsorption of approximately 13.3 mg/g. Ning et al. [33] used silica-based isoBu-BTP/SiO_2_-P adsorbent to separate Ru, Rh, and Pd from nitrate solution, and their saturated adsorption capacities reached 0.37 mmol/g, 0.34 mmol/g, and 0.71 mmol/g, respectively. Su et al. [34] prepared a silica-based polymer adsorbent (isopentyl-BTBP/SiO_2_-P) by a maceration method in a vacuum for efficient and continuous separation of Pd from complex systems. In a HNO_3_ solution of 3 M, the distribution coefficient of Pd was 5226 mL/g, and the maximum adsorption capacity was 31.7 mg/g.

Considering the advantages of NTAamide(C8) extractant and SiO_2_-P carrier, this study aims to combine them to prepare a new adsorption material, which is expected to achieve high selectivity and adsorption percentage simultaneously. The adsorption performance and mechanism of recovery of palladium ion from simulated HLLW were investigated.

## 2. Materials and Methods

### 2.1. Chemicals

Nitric acid was supplied by Sinopharm Chemical Reagent Co., Ltd. (Beijing, China). Rhodium nitrate was provided by Kunming Bosen Precious Metal Materials Processing Co., Ltd. (Kunming, China). The standard solutions of Gd, Ho, Dy, Ce, Sn, Re, Sm, Pr, Tb, Mo, and Sr were obtained from Guobiao Testing & Certification Co., Ltd. (Beijing, China). All chemicals were of analytical grade. Deionized water was obtained through a Smart-Q15 system (Hitech Instruments Co., Ltd. Shanghai, China) in the lab. Palladium nitrate dihydrate, ruthenium nitrosyl nitrate, and other chemicals were purchased from Shanghai Macklin Biochemical Co., Ltd. (Shanghai, China). All chemicals were analytical grade.

### 2.2. Material Preparation

#### 2.2.1. Preparation of SiO_2_-P

**Figure 2:**
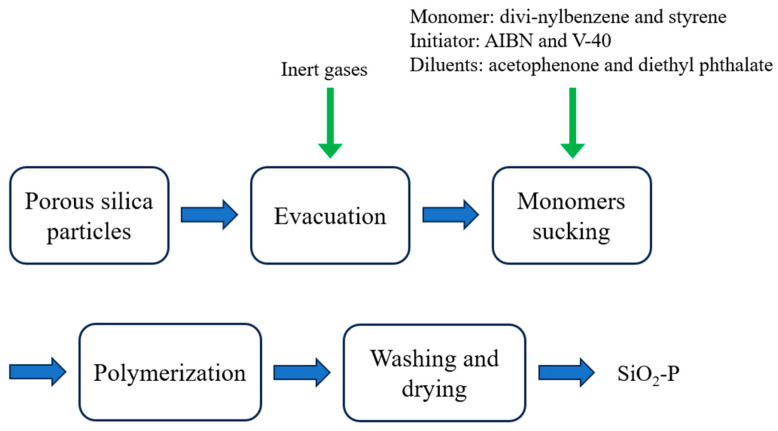
Preparatory flow sheet of SiO_2_-P.

The inorganic organic composite carrier SiO_2_-P was prepared by an in situ polymerization method [35,36]. The preparatory flow sheet of the SiO_2_-P is illustrated in Figure 2. First, the stabilizers in styrene and divinylbenzene were removed by vacuum distillation and washed with a dilute alkali solution, respectively. Porous SiO_2_ was added to the round-bottom flask of a rotary evaporator, and the flask was rotated at 40 rpm. The air in the flask was displaced by first vacuuming and then purging with inert gases. The air pressure was reduced to 3 kPa again. Acetophenone and diethyl phthalate were used as diluents and placed in a beaker. Then, the monomers (divinylbenzene and styrene) were added to the beaker and mixed thoroughly, followed by adding initiator AIBN and V-40. Then, the mixture was poured into the round-bottom flask to fully mix with SiO_2_. Inert gas was introduced to ensure that the device is in an oxygen-free environment. The co-polymerization reaction started between styrene and divinylbenzene upon heating. After the reaction was completed, the product SiO_2_-P was washed and dried before use.

#### 2.2.2. Preparation of NTAamide(C8)/SiO_2_-P

**Figure 3:**
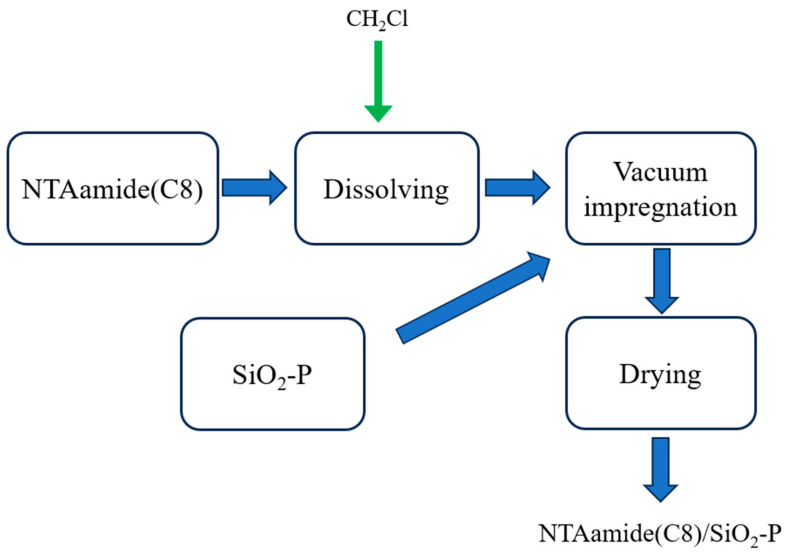
Preparatory flow sheet of NTAamide(C8)/SiO_2_-P.

NTAamide(C8) ligand was loaded onto the SiO_2_-P carrier by the impregnation method in a vacuum [37,38,39]. The preparatory flow sheet of the NTAamide(C8)/SiO_2_-P is illustrated in Figure 3. First, an appropriate amount of methanol was added to SiO_2_-P and mixed thoroughly, followed by solid–liquid separation. The above steps were repeated three times, and then the obtained SiO_2_-P was dried. SiO_2_-P (10 g) was placed in a round-bottom flask. Then, 5 g of NTAamide(C8) was dissolved in an appropriate amount of dichloromethane and transferred to the round-bottom flask. The flask was rotated at 100 rpm on a rotary evaporator at room temperature and pressure for 1 h, and then left standing for 1 h. The flask was subsequently heated in a water bath to 310 K while maintaining the rotation speed at 100 rpm for 1.5 h before turning on the cooling water circulation device. After the above steps, the vacuum pump was turned on to slowly depressurize the extractant into the channel of SiO_2_-P until the diluent dichloromethane was completely volatilized. After drying, the NTAamide(C8)/SiO_2_-P was obtained.

### 2.3. Material Characterization

The morphology and composition of the composites were measured by scanning electron microscopy (SEM, Hitachi S4800, Hitachi Ltd., Tokyo, Japan) equipped with an energy dispersive spectrometer (EDS). Thermogravimetric (TG) analysis of the composites was conducted using a thermal analyzer (TGA/DSC3+, Mettler-Toledo International Inc., Zurich, Switzerland). The structural parameters were measured using the Brunauer–Emmett–Teller method (BET; ASAP2460, Micromeritics instrument (Shanghai) Ltd., Shanghai, China). X-ray diffraction (XRD) was performed on a diffractometer (Ultima IV, Rigaku Beijing Corporation, Beijing, China). An X-ray photoelectron spectrometer (XPS; ESCALAB 250, Thermo Fisher Scientific, Waltham, MA, USA) was used to characterize the surface properties. Fourier transform–infrared spectroscopy (FT-IR) spectra were collected on an FT-IR spectrometer (Nicolet 6700, Thermo Fisher Scientific, Waltham, MA, USA). The content of metal ions in the solution was measured by an inductively coupled plasma emission spectrometer (ICP-OES; JY2000-2, Horiba instruments (Shanghai) Co., Ltd., Shanghai, China).

### 2.4. Adsorption Experiments

A certain amount of the adsorbent was mixed with the metal ion solutions (0.01–1 M) in a centrifuge tube, which was placed in a constant-temperature water bath and oscillated at 280 rpm. After the adsorption reached equilibrium, the mixture was centrifuged and the supernatant was used to analyze the concentration of metal ions by ICP-OES. The acidity of the solution, the dosage of the adsorbent, the concentration of metal ions, the temperature, and the coexisting ions were adjusted to evaluate the static adsorption performance of the adsorption material. Several important physical parameters such as adsorption capacity (Q, mg/g), adsorption percentage (E), distribution coefficient (K_d_, mL/g), and separation factor (SF) are defined as follows:

(1) $$Q=\frac{(C_{0}-C)\cdot V}{m}$$

(2) $$E=\frac{(C_{0}-C)\cdot V}{C_{0}}\times 100\%$$

(3) $$K_{d}=\frac{\left(C_{0}-C\right)}{C_{0}}\cdot \frac{V}{m}$$

(4) $$SF_{A/B}=\frac{K_{dA}}{K_{dB}}$$

where C_0_ and C (mg/L) represent the initial and equilibrium metal ion concentrations, respectively, m (g) is the mass of the adsorbent, and V (mL) is the volume of the aqueous phase. A and B represent two different components in the same aqueous phase.

## 3. Results and Discussions

### 3.1. Characterization

**Figure 4:**
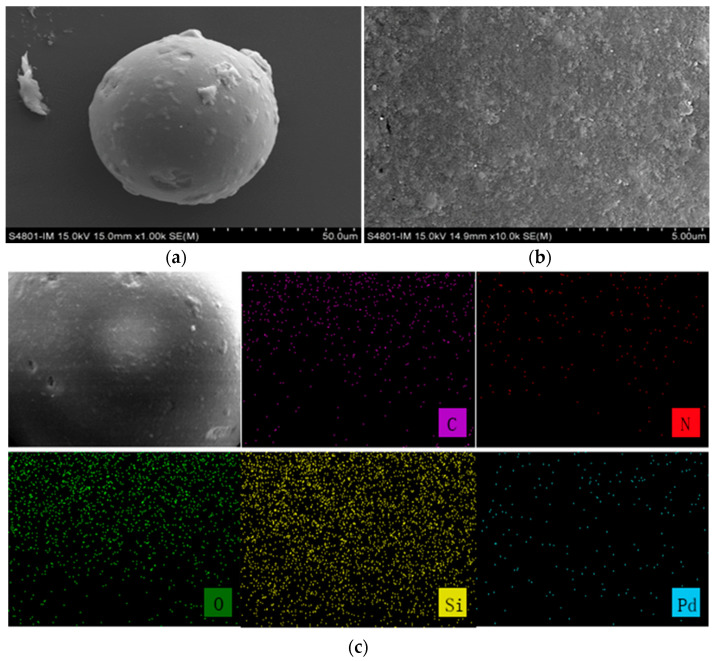
(**a**,**b**) SEM images of NTAamide(C8)/SiO_2_-P; (**c**) SEM-EDS images of Pd-NTAamide(C8)/SiO_2_-P.

To determine the morphology and elemental mapping of the adsorbent, SEM characterization was performed. The SEM results of NTAamide(C8)/SiO_2_-P and the SEM-EDS results of Pd-loaded NTAamide(C8)/SiO_2_-P are shown in Figure 4. The surface of the NTAamide(C8)/SiO_2_-P sphere is relatively rough, which indicates that NTAamide(C8) entered the inner pores of SiO_2_-P. The uniform distribution of N elements indicates that NTAamide(C8) was uniformly loaded onto SiO_2_-P. The results after adsorption show that Pd elements are evenly distributed on the surface of the sphere, confirming the adsorption of Pd.

The SEM results of SiO_2_-P are shown in Figure S1. In addition, the organic content of NTAamide(C8) in NTAamide(C8)/SiO_2_-P was determined to be 35.63% through a TG thermal analyzer (Figure S2). By analyzing N_2_-adsorption–desorption isotherm and pore diameter distribution, it was calculated that the BET surface area is 26.4 m^2^/g, pore volume is 0.17 cm^3^/g, and the average pore diameter is 29.4 nm (Figure S4 and Table S1).

### 3.2. Adsorption Experiments

#### 3.2.1. Effect of HNO_3_ Concentration

**Figure 5:**
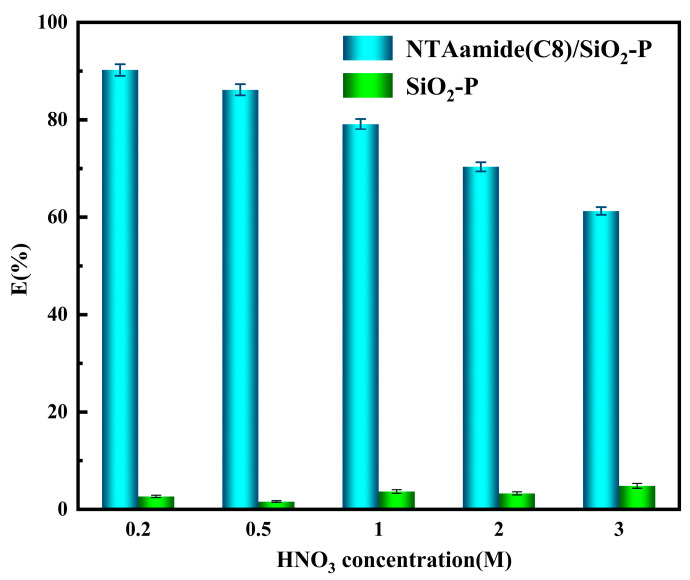
Effects of HNO_3_ concentration on the adsorption percentage of Pd(II) onto NTAamide(C8)/SiO_2_-P and SiO_2_-P. (C_0_: 1 mM, *m*/*v*: 0.05 g/10 mL, Temperature: 298 K, and r: 280 rpm).

The effects of HNO_3_ concentration on the adsorption of Pd(II) by NTAamide(C8)/SiO_2_-P and SiO_2_-P were studied (Equation (2)). As shown in Figure 5, NTAamide(C8)/SiO_2_-P exhibits a strong affinity for Pd(II) at a low acid concentration (0.2 M). The adsorption of Pd(II) decreases with the increasing HNO_3_ concentration. At a high HNO_3_ concentration, the protonated N donor with positive charges tend to repel Pd(II) ions, significantly reducing the adsorption percentage. Compared with carrier SiO_2_-P, the adsorption capacity of NTAamide(C8)/SiO_2_-P was greatly increased. The experiments of NTAamide(C8)/SiO_2_-P were repeated three times with an error of less than 1.5%.

#### 3.2.2. Effect of NTAamide(C8)/SiO_2_-P Dosage

**Figure 6:**
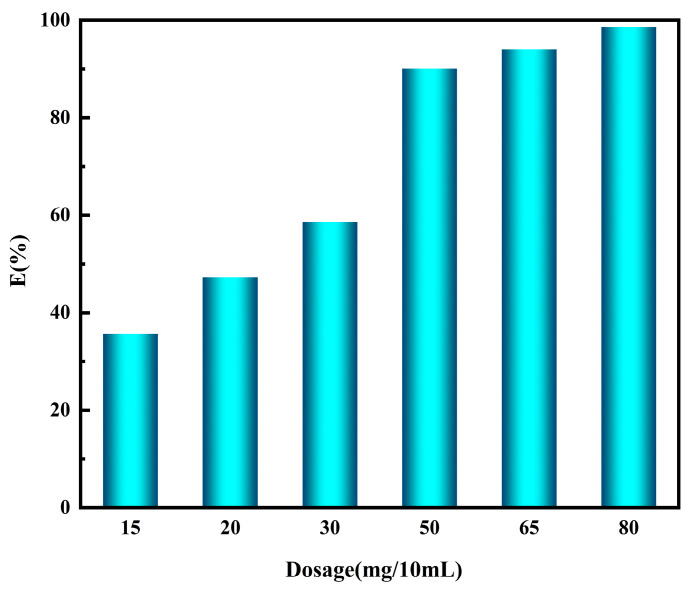
Effect of NTAamide(C8)/SiO_2_-P dosage on the adsorption percentage of Pd(II). (C_0_: 1 mM, C_HNO3_: 0.2 M, Temperature: 298 K, and r: 280 rpm).

As shown in Figure 6, with the increasing dosages of NTAamide(C8)/SiO_2_-P, the adsorption percentage of Pd increases. When 80 mg of NTAamide(C8)/SiO_2_-P is added, the adsorption percentage reaches 98.41% (Equation (2)). More adsorbents provide more adsorption sites for Pd(II), resulting in a lower equilibrium concentration and higher adsorption percentage.

#### 3.2.3. Selectivity of NTAamide(C8)/SiO_2_-P

**Figure 7:**
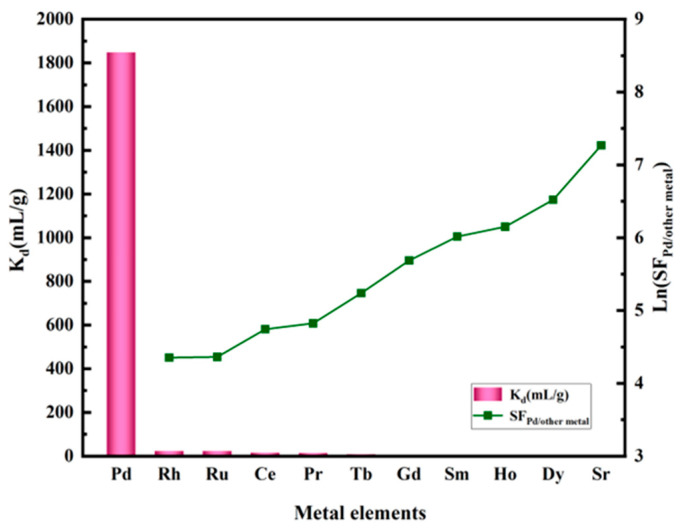
Adsorption of 11 typical metal ions onto NTAamide(C8)/SiO_2_-P (C_0_: 1 mM, C_HNO3_: 0.2 M, *m*/*v*: 0.05 g/10 mL, Temperature: 298 K, and r: 280 rpm).

The adsorption selectivity of NTAamide(C8)/SiO_2_-P was studied (Figure 7). In 0.2 M nitric acid, NTAamide(C8)/SiO_2_-P has no obvious adsorption effect on 10 metal ions. Comparatively, the distribution coefficient of Pd (Equation (3)) is significantly higher, suggesting that NTAamide(C8)/SiO_2_-P has good adsorption selectivity (SF_Pd/other metals_ > 77.8 (Equation (4)) and can be used to separate Pd from other metal ions. NTAamide(C8)/SiO_2_-P was compared with two similar materials. Me_2_-CA-BTP/SiO_2_-P [37] is a novel silica-based adsorbent, which is prepared to separate minor actinides from fission products in HLLW. The K_d_ of Pd is below 100 mL/g and SF_Am/Pd_ value is 17 in 0.1 M HNO_3_. IsoBu-BTP/SiO_2_-P [33] has good adsorption capacity for Pd, but also adsorbs a large amount of Ru and Rh. It is unable to selectively separate Pd. Compared with these adsorptions, NTAamide(C8)/SiO_2_-P has superiority in selectivity. NTAamide(C8) has nitrogen donor atoms at the center of the backbone. In the HSAB (Hard-Soft-Acid-Base) framework, NTAamide(C8) is classified as a soft base. Among these metal ions, Pd is the only soft acid with good electron acceptance ability. Pd(II) can coordinate with the ligands through nitrogen donor atoms on the central frame and carbonyl groups.

#### 3.2.4. Effect of NaNO_3_ Concentration on Adsorption Performance

**Figure 8:**
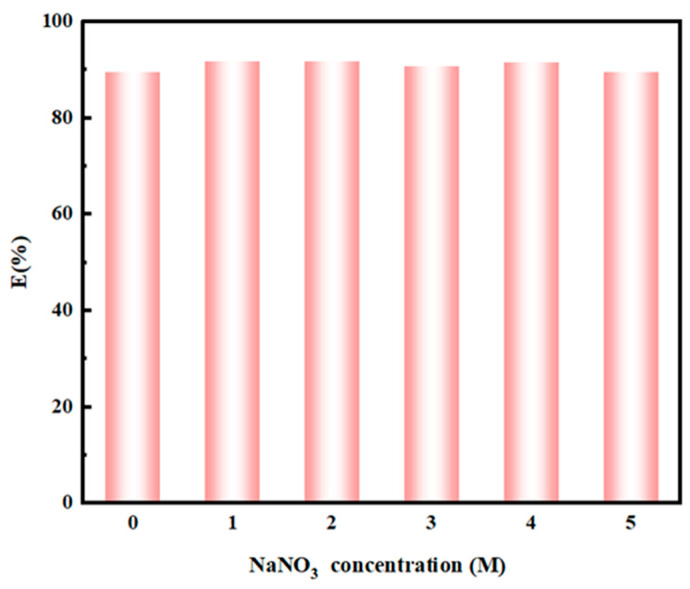
Effect of NaNO_3_ concentration on the adsorption of Pd(II) onto NTAamide(C8)/SiO_2_-P (C_0_: 1 mM, C_HNO3_: 0.2 M, *m*/*v*: 0.05 g/10 mL, temperature: 298 K, and r: 280 rpm).

During the adsorption process, the N donor is protonated, requiring nitrate ions to maintain the charge balance. Therefore, the effect of sodium nitrate on the adsorption was investigated. The concentration of NaNO_3_ varies from 0 to 5 M, and the adsorption of Pd(II) by NTAamide(C8)/SiO_2_-P was studied in 0.2 M nitric acid (Figure 8). The system without NaNO_3_ can achieve a high adsorption percentage. The presence of NaNO_3_ slightly improves the adsorption percentage of NTAamide(C8)/SiO_2_-P. The highest adsorption percentage (92.4%) is achieved when the NaNO_3_ concentration is 2 M, which is about 2.2% higher than the system without NaNO_3_. Considering that NaNO_3_ has a negligible effect on adsorption, the system without NaNO_3_ is used for subsequent experiments.

#### 3.2.5. Adsorption Kinetics

The kinetics of Pd(II) adsorption on NTAamide(C8)/SiO_2_-P was studied at 298 K. Pseudo-first order kinetics (Equation (5)) and pseudo-second order kinetics (Equation (6)) were used to fit the curves of adsorption capacity vs. time.

(5) $$\ln(Q_{e}-Q_{t})=\ln Q_{e}-k_{1}\cdot t$$

(6) $$\frac{t}{Q_{t}}=\frac{t}{Q_{e}}+\frac{1}{k_{2}\cdot {Q_{e}}^{2}}$$

where Q_e_ and Q_t_ (mg/g) (Equation (1)) represent the equilibrium adsorption capacity and adsorption capacity at time t(min), respectively; k_1_ (min^−1^) and k_2_ (mg/(g·min)) represent the adsorption rate constants of pseudo-first and pseudo-second order kinetics, respectively.

Their non-linear form is described as Equations (7) and (8), respectively:

(7) $$Q_{t}=Q_{e}\left(1-e^{-k_{1}\cdot t}\right)$$

(8) $$Q_{t}=\frac{k_{2}\cdot {Q_{e}}^{2}\cdot t}{1+k_{2}\cdot Q_{e}\cdot t}$$

**Figure 9:**
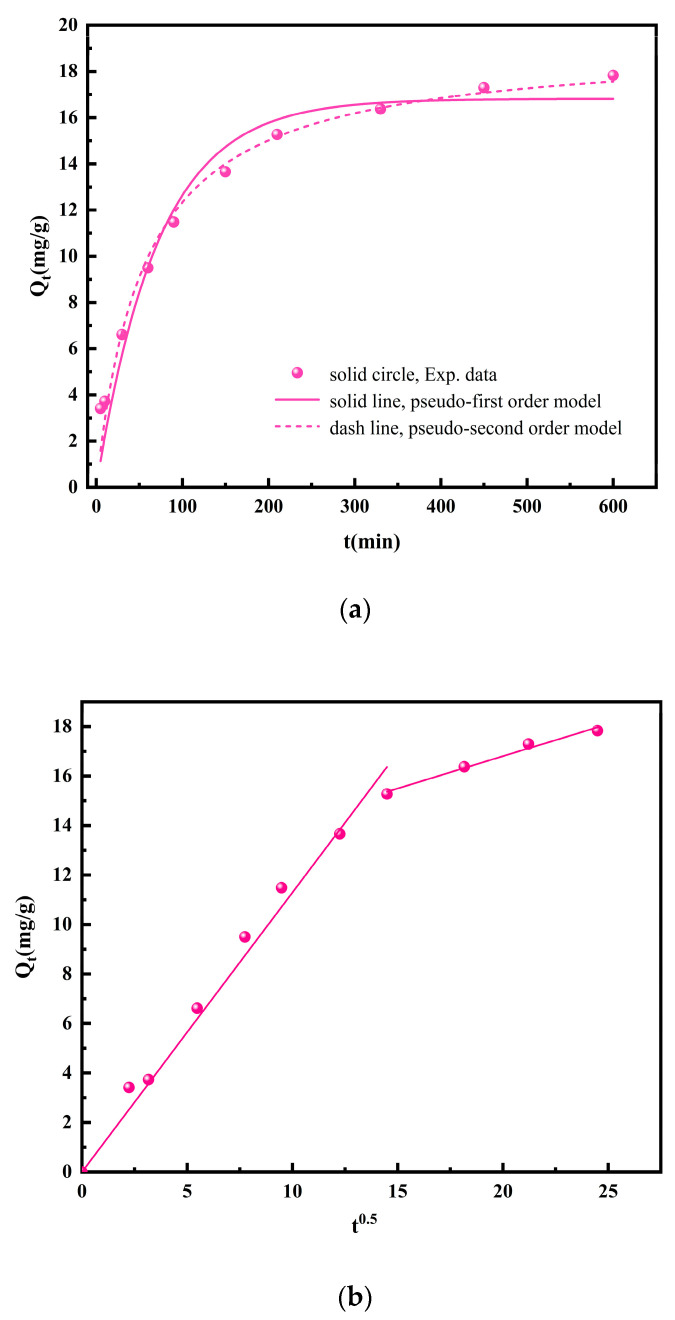
(**a**) Non-linear pseudo-first order, pseudo-second order kinetic, and (**b**) intraparticle diffusion model fitting for Pd(II) adsorption onto NTAamide(C8)/SiO_2_-P. (C_0_: 1 mM, C_HNO3_: 0.2 M, *m*/*v*: 0.05 g/10 mL, Temperature: 298 K, and r: 280 rpm).

As shown in Figure 9a and Table 1, the pseudo-second order model has a higher correlation coefficient (R^2^), and its theoretical equilibrium adsorption capacity (Q_e_) is closer to the experimental value (Q_e.exp_), suggesting it is more suitable to describe the adsorption process. The correlation coefficient was fitted through the formula. It indicates the degree of agreement between the experimental data and the model.

In order to explain the diffusion mechanism, the intraparticle diffusion model (Equation (9)) was applied to the experimental data [40]:

(9) $$Q_{t}=k_{id}t^{0.5}+C$$

where k_id_ (mg/(g·min^0.5^)) represents the intraparticle diffusion rate constant and the value of C is affected by boundary layer thickness.

As shown in Figure 9b and Table 1, the plots present two steps. The first step was surface diffusion stage and the second step was intraparticle diffusion stage. The plot did not pass through the origin, indicating that intraparticle diffusion is not the only rate-determining step. The decrease in slope indicates that the adsorption sites were occupied, and the adsorption rate decreased. The adsorption gradually reached equilibrium.

#### 3.2.6. Adsorption Isotherm

**Figure 10:**
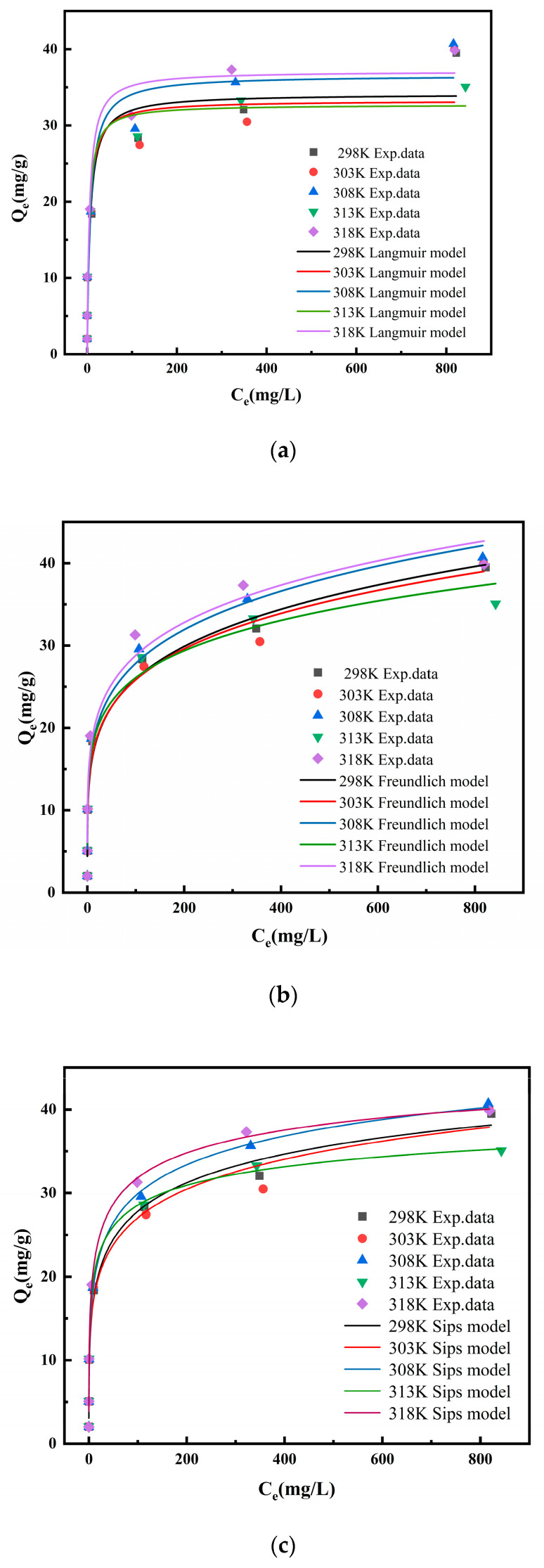
(**a**) Langmuir, (**b**) Freundlich, and (**c**) Sips isotherm fitting for Pd(II) adsorption onto NTAamide(C8)/SiO_2_-P. (C_HNO3_: 0.2 M, *m*/*v*: 0.05 g/10 mL, and r: 280 rpm).

By changing the initial concentration of Pd in the solution, the adsorption isotherm at 298 K, 303 K, 308 K, 313 K, and 318 K were measured. Five adsorption isotherm models (Langmuir, Freundlich, Temkin, Dubinin–Radushkevich, and Sips (Equations (10)–(14))) were used to fit the experimental data to understand the relationship between adsorption capacity (Q) and Pd equilibrium concentration (Figure 10 and Table 2). From Table 2, the R^2^ of the Sips isotherm model is higher than those of the other four isotherm models. Therefore, the Sips isotherm model is more suitable for describing the adsorption behavior of Pd(II) on NTAamide(C8)/SiO_2_-P. The Sips isotherm is a combination of the Langmuir and Freundlich isotherm models, and it is based on the Freundlich equation assumption [40]. It is speculated that the adsorption of palladium is through a multi-molecular layer chemical process. 1/n is the Sips isotherm exponent, which can be used to indicate the non-uniformity of adsorption sites. If 1/n < 1, the adsorption sites on the adsorbent surface are not uniformly distributed. The Sips isotherm model also indicates a finite limit on adsorption capacity. The theoretical saturated adsorption capacity of NTAamide(C8)/SiO_2_-P is higher than that of a similar material IONP@SiO_2_ (6.5 mg/g) [41].

(10) $$Q_{e}=\frac{Q_{m}\cdot K_{L}\cdot C_{e}}{1+K_{L}\cdot C_{e}}$$

(11) $$Q_{e}=K_{F}\cdot {C_{e}}^{\frac{1}{n}}$$

(12) $$Q_{e}=BlnA+BlnC_{e}$$

(13) $$Q_{e}=Q_{m}exp\left(-K_{DR}\varepsilon^{2}\right)$$

(14) $$Q_{e}=\frac{Q_{m}{\left(K_{s}\cdot C_{e}\right)}^{\frac{1}{n}}}{1+{\left(K_{s}\cdot C_{e}\right)}^{\frac{1}{n}}}$$

where Q_e_ and Q_m_ (mg/g) (Equation (1)) represent the equilibrium and theoretical saturated adsorption capacity, respectively, K_L_ (L/mg), K_F_ (mg^1–n^·L^n^/g), K_DR_ (mol^2^/kJ^−2^), and K_S_ (mg^1/n^/L^1/n^) represent the model constants of Langmuir, Freundlich, Dubinin–Radushkevich, and Sips adsorption isotherm models, respectively, and n is adsorption intensity. A and B are Temkin constants, B = RT/b. Polanyi potential ε = RTln(1 + 1/C_e_).

#### 3.2.7. Adsorption Thermodynamics

To understand the thermodynamics of NTAamide(C8)/SiO_2_-P adsorption and clarify the relationship between the adsorption and temperature, the adsorption isotherm experiments were conducted at 298–318 K, and the thermodynamic calculation was carried out by the Van’t Hoff equation (Equations (15) and (16)).

(15) $$\ln K_{F}=-\frac{\Delta H^{\theta }}{RT}+\frac{\Delta S^{\theta }}{R}$$

(16) $$\Delta G^{\theta }=\Delta H^{\theta }-\Delta S^{\theta }\cdot T$$

where K_F_ is the model constant of the Freundlich adsorption isotherm model. ΔG^θ^, ΔH^θ^, and ΔS^θ^ are the changes in Gibbs free energy (J/mol), enthalpy (J/mol), and entropy (J/(K·mol)), respectively. R is the universal gas constant (8.314 J/(K·mol)).

Although the R^2^ of the Sips isotherm model is higher, the deviation of Ks is relatively large. The Sips isotherm model is based on the Freundlich equation assumption, and the R^2^ value of the Freundlich isotherm model is close to that of Sips. Therefore, K_F_ of the Freundlich adsorption isotherm model was used for the thermodynamic calculation.

**Figure 11:**
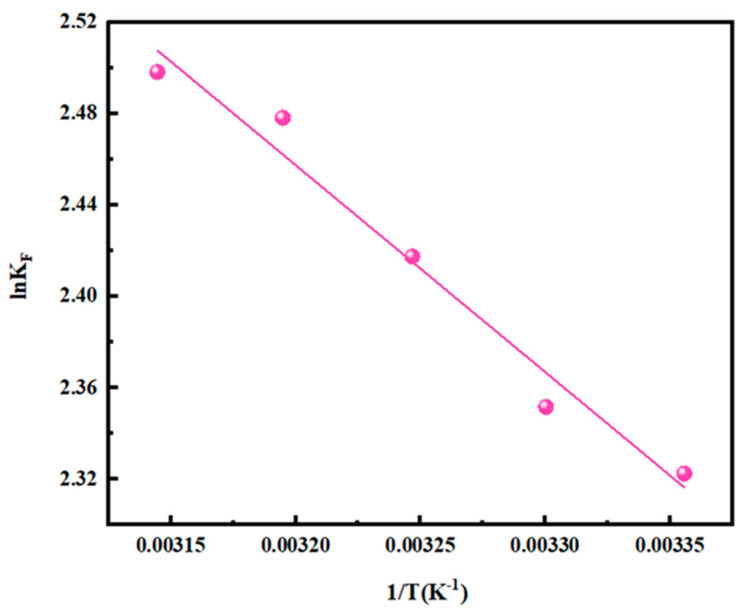
Thermodynamics fitting for Pd(II) adsorption onto NTAamide(C8)/SiO_2_-P.

The calculation results are shown in Figure 11 and Table 3. A positive ΔH value indicates that the adsorption of Pd(II) is an endothermic reaction, which is consistent with the adsorption isotherm experiments (the equilibrium adsorption capacity increases with the temperature). The ΔG value remains negative at different temperatures, indicating that the adsorption of NTAamide(C8)/SiO_2_-P is a spontaneous process. The ΔS value is 44.5 J/K·mol for the adsorption process, suggesting that the adsorption is driven by entropy.

### 3.3. Adsorption Mechanism

The interaction mechanism of NTAamide(C8)/SiO_2_-P was studied using XPS and FT-IR.

**Figure 12:**
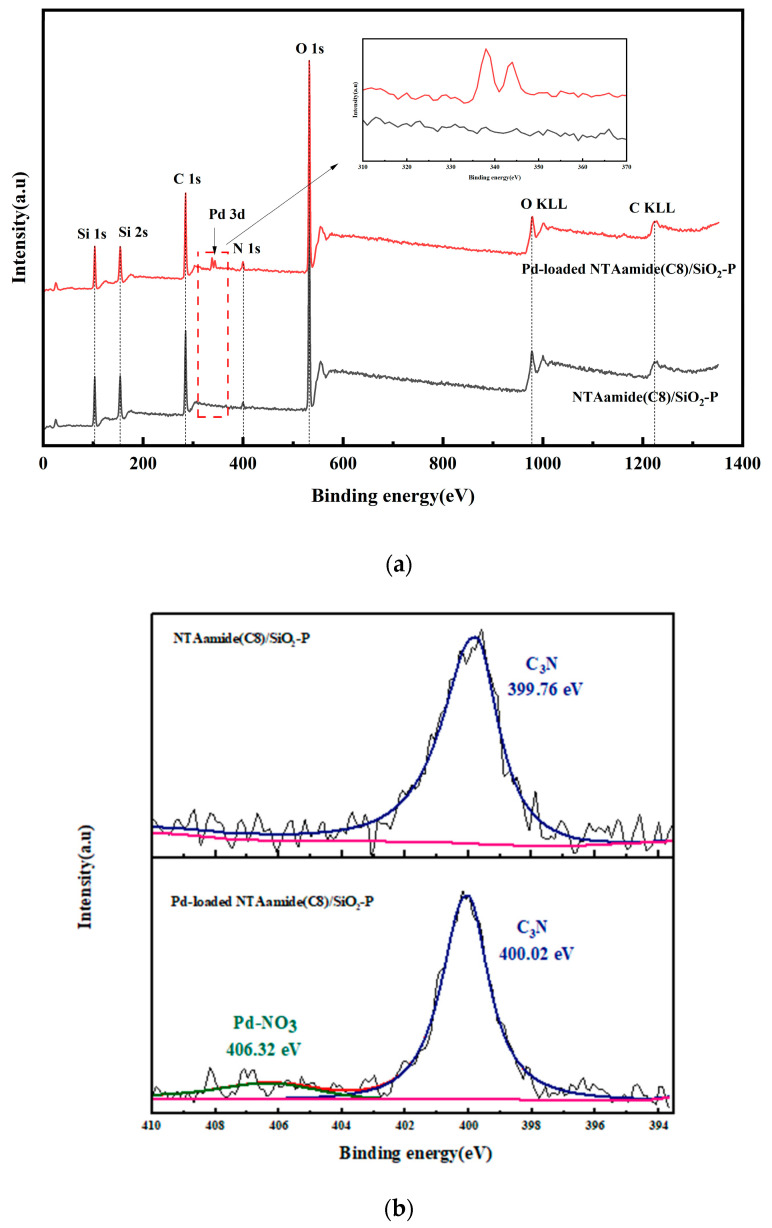
(**a**) XPS spectra of NTAamide(C8)/SiO_2_-P and Pd-loaded NTAamide(C8)/SiO_2_-P, (**b**) high resolution scans of N 1s spectrum before and after adsorption.

The XPS spectra of NTAamide(C8)/SiO_2_-P and Pd-loaded NTAamide(C8)/SiO_2_-P are shown in Figure 12a. The presence of C1s, N1s, and O1s peaks and the two Auger peaks of C and O in the spectrum is consistent with the composition of NTAamide(C8)/SiO_2_-P. The N1s peak emerges at 400 eV, indicating that NTAamide(C8) has been loaded onto SiO_2_-P. A characteristic peak of Pd3d is found at 338 eV after adsorption, indicating that Pd has been adsorbed by NTAamide(C8)/SiO_2_-P. The XPS spectra of N1s is shown in Figure 12b. For NTAamide(C8)/SiO_2_-P and Pd-loaded NTAamide(C8)/SiO_2_-P, the binding energy of C_3_N are 399.76 eV and 400.02 eV, respectively. The change in binding energy of C_3_N implies that it may play an important role in the adsorption behavior. The specific mechanism will be further studied through FT-IR.

**Figure 13:**
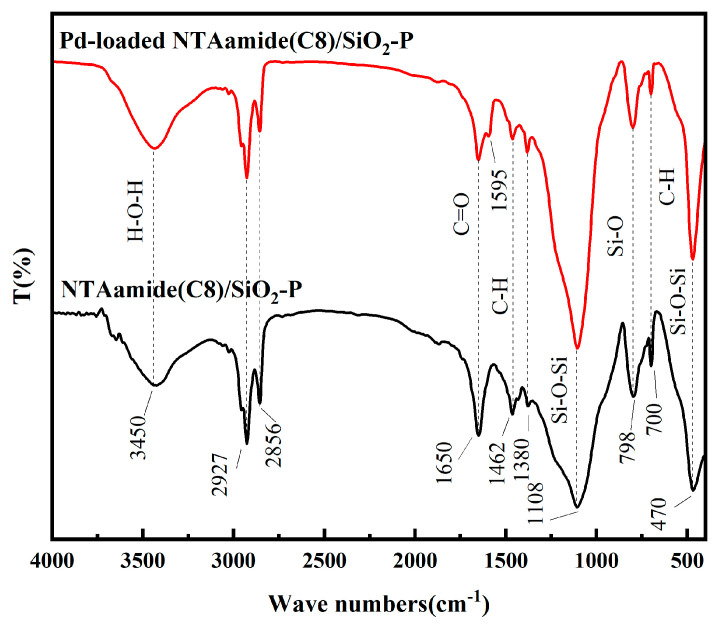
FT-IR spectra of NTAamide(C8)/SiO_2_-P and Pd-loaded NTAamide(C8)/SiO_2_-P.

The FT-IR spectra of NTAamide(C8)/SiO_2_-P and Pd-loaded NTAamide(C8)/SiO_2_-P are shown in Figure 13. The characteristic peak at 798cm^−1^ corresponds to the stretching vibration of Si-O, while the peaks at 470 and 1108 cm^−1^ are associated with the stretching vibrations of Si-O-Si. The out-of-plane deformation vibration of the aromatic hydrocarbon (=C-H) is observed at 700 and 1462 cm^−1^. The characteristic peaks at 1380 cm^−1^ and 1650 cm^−1^ correspond to the deformation vibration of the methyl group (CH_3_) and the stretching vibration of C=O in NTAamide(C8). The stretching vibration peaks of methylene (C-H) in NTAamide(C8) appear at 2856 and 2927 cm^−1^. The wide peak at 3450 cm^−1^ is due to the vibration of H-O-H in water that has not been completely removed. NTAamide(C8) contains a tertiary amine structure (R_3_N), which exhibits double peaks at 1230~1030 cm^−1^. However, due to the stronger characteristic peak of Si-O-Si at 1108 cm^−1^, the double peaks become a wider peak. After adsorption, the characteristic peaks are slenderer, indicating that the tertiary amine structure may coordinate with Pd(II), which is consistent with the composition of NTAamide(C8)/SiO_2_-P. According to the relative literature [22,42], the 2:1 ligand/Pd^2+^ complexes were formed in a low concentration of HNO_3_ solution. Two NTAamide(C8) ligand molecules coordinated with one four-coordinated Pd^2+^ via one of the three carbonyl groups and the central N atom, forming a complex cation. There are two NO^3 −^ ions anion as the counterpart ions for charge balance, and the NO^3−^ ions only have a salting-out effect on the ligands under this condition [22]. The single peak originally located at 1650 cm^−1^ was split into two peaks at 1650 and 1595 cm^−1^, which is also consistent with the adsorption mechanism.

NTAamide(C8) has nitrogen donor atoms at the center of the backbone. In the HSAB (Hard-Soft-Acid-Base) framework, NTAamide(C8) is classified as a soft base. Pd(II) can coordinate with the extractant through nitrogen donor atoms on the central frame and carbonyl groups. NO_3_^−^ is required to participate in the reaction to maintain charge balance.

### 3.4. Reusability

**Figure 14:**
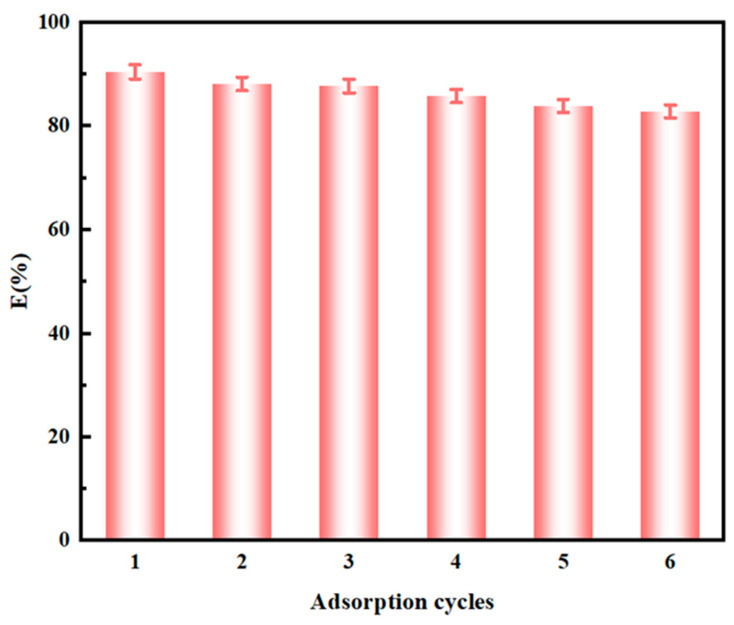
The reusability of NTAamide(C8)/SiO_2_-P(C_0_: 1 mM, C_HNO3_: 0.2 M, *m*/*v*: 0.05 g/10 mL, Temperature: 298 K, and r: 280 rpm).

Reusability is an important factor in evaluating the performance of adsorbents. The reusability of NTAamide(C8)/SiO_2_-P was studied through six cycles of adsorption and desorption. First, 50 mg of the adsorbent was added to the Pd solution to reach adsorption equilibrium under certain conditions, and then the solution was removed. After drying, the used adsorbent was desorbed in a mixed solution of thiourea (0.01 M) and nitric acid (0.1 M) at 298 K while shaking at 280 rpm. As shown in Figure 14, the adsorption percentage after six adsorption–desorption cycles was 82.92% (a decrease of approximately 7.3% from the original value). The solid–liquid separation operation during the experiment may result in a slight loss of the adsorbent, which can lead to a decrease in adsorption percentage. Therefore, the NTAamide(C8)/SiO_2_-P adsorbent has good reusability, making the adsorption process economically feasible. The experiments were repeated three times with an error of less than 2.2%.

## 4. Conclusions

In this research, a mesoporous silica-based composite, NTAamide(C8)/SiO_2_-P, was prepared by the impregnation method in a vacuum. The resulting NTAamide(C8)/SiO_2_-P could adsorb Pd(II) efficiently in a nitric acid system. The results indicate that low acidity is conducive to the adsorption of Pd(II), with the uptake rate over 90%. In 0.2 M HNO_3_ solution, NTAamide(C8)/SiO_2_-P exhibits excellent adsorption selectivity for Pd(II) (SF_Pd/other metals_ > 77.8). The adsorption thermodynamics study has shown that the adsorption of Pd(II) on NTAmide(C8)/SiO_2_-P is a spontaneous and endothermic process. Its adsorption kinetics matched well with the pseudo-second order kinetics model and the adsorption isotherm data met well with Sips model. After adsorption, thiourea desorption can be used to separate Pd(II), making NTAamide(C8)/SiO_2_-P an excellent material for recovering Pd(II) from HLLW. It indicates the potential practicability of NTAamide(C8)/SiO_2_-P in application. Finally, XPS and FT-IR results revealed the coordination mechanism in Pd adsorption. NTAamide(C8)/SiO_2_-P has excellent adsorption selectivity and capacity and excellent reusability, meaning it has great application prospects in Pd separation. pH has a significant impact on the adsorption performance of NTAamide(C8)/SiO_2_-P, and it is expected to be improved in the future. Although SiO_2_-P is an excellent carrier, there are many other carriers that may contain a larger specific surface area or larger loading capacity. Replacing the carrier may improve the performance of the adsorbent.

## Figures and Tables

**Table 1:** Kinetics parameters of Pd(II) adsorption on NTAamide(C8)/SiO_2_-P.

Pseudo-First-Order Model			Pseudo-Second-Order Model			Intraparticle Diffusion Kinetic Model		
K_1_	Q_e_	R^2^	K_2_	Q_e_	R^2^	K_id_	C	R^2^
0.0139	16.8	0.957	9.38 × 10^−4^	19.2	0.982	0.261	11.6	0.983

**Table 2:** Isotherm parameters of Pd(II) adsorption on NTAamide(C8)/SiO_2_-P.

T	Langmuir Isotherm				Freundlich Isotherm		
	K_L_	Q_m_		R^2^	K_F_	n	R^2^
298 K	0.150	34.1		0.903	10.2	4.93	0.985
303 K	0.185	33.3		0.870	10.5	5.11	0.977
308 K	0.136	36.6		0.902	11.2	5.07	0.985
313 K	0.222	32.7		0.901	11.9	5.87	0.973
318 K	0.184	37.1		0.913	12.2	5.34	0.965
	**Temkin isotherm**				**D-R model**		
	**A**	**B**		**R^2^**	**K_DR_**	**Q_m_**	**R^2^**
298 K	41.8	3.43		0.965	7.12 × 10^−8^	25.3	0.762
303 K	54.1	3.32		0.966	3.07 × 10^−8^	24.5	0.736
308 K	50.7	3.61		0.983	4.09 × 10^−8^	27.1	0.788
313 K	149.2	2.95		0.984	1.56 × 10^−8^	24.3	0.728
318 K	61.29	3.63		0.974	2.14 × 10^−8^	27.1	0.770
	**Sips isotherm**						
	**K_S_**	**Q_m_**	**n**	**R^2^**			
298 K	4.62 × 10^−3^	63.2	3.19	0.993			
303 K	5.73 × 10^−4^	84.0	3.84	0.980			
308 K	6.22 × 10^−3^	64.6	3.22	0.995			
313 K	4.40 × 10^−2^	46.1	3.06	0.994			
318 K	3.54 × 10^−2^	52.1	2.80	0.987			

**Table 3:** Adsorption thermodynamic parameters of NTAamide(C8)/SiO_2_-P. (R^2^ = 0.974).

ΔH (J/mol)	ΔS(J/(K·mol))	ΔG(J/mol)				
		298 K	303 K	308 K	313 K	318 K
7.53 × 10^3^	44.5	−5.73 × 10^3^	−5.95 × 10^3^	−6.18 × 10^3^	−6.40 × 10^3^	−6.62 × 10^3^

## Data Availability

Data are contained within the article.

## Supplementary Materials

The following supporting information can be downloaded at: https://www.mdpi.com/article/10.3390/nano14060544/s1.
