# Supplementary material for: Efficient and Selective Removal of Palladium from Simulated High-Level Liquid Waste Using a Silica-Based Adsorbent NTAamide(C8)/SiO2-P
Source: Nanomaterials (Basel). 2024 Mar 20;14(6):544. doi: 10.3390/nano14060544 (PMC10974362; doi:10.3390/nano14060544)
Supplement: Supplementary file 1 [file nanomaterials-14-00544-s001.zip › nanomaterials-2871090-supplementary.pdf]

Supporting Information

Efficient and Selective Removal of Palladium from Simulated High-Level Liquid Waste Using a Silica-Based Adsorbent NTAamide(C8)/SiO_2-P

Jiaxuan Shi [1], Junli Wang [1,2], Wentao Wang [2], Xuan Wu [2], Hui Wang [2,*] and Jianwei Li [1,*]

[1] State Key Laboratory of Chemical Resource Engineering, College of Chemical Engineering, Beijing University of Chemical Technology, Beijing 100029, China
[2] Department of Radiochemistry, China Institute of Atomic Energy, Beijing 102413, China
* Correspondence: hwihwi@126.com (H.W.); lijw@mail.buct.edu.cn (J.L.)

3. Results

3.1. Characterization

To determine the morphology of the carrier, SEM characterization was performed. The SEM results of SiO_2-P are shown in Figure S1. The surface of /SiO_2-P sphere is relatively rough, ensuring its high specific surface area. It is beneficial for loading more ligands.

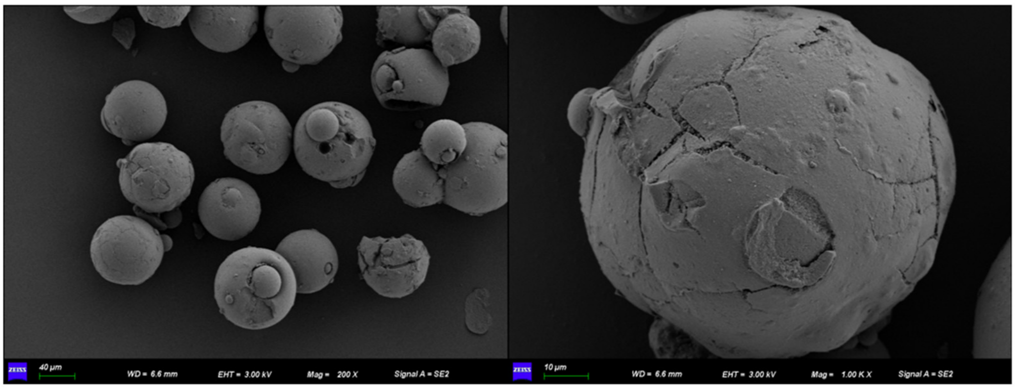

Figure. S1 SEM images of SiO_2-P.

To determine the organic content in NTAamide(C8)/SiO_2-P, a TG thermal analyzer was used to test the decomposition curve under a nitrogen atmosphere with a heating rate of 5 K/min from room temperature to 1073 K. As shown in Figure S2, the TG curve shows continuous weight loss as the test temperature increases. The weight loss of NTAamide (C8)/SiO_2-P (42.76%) starts at around 413 K and completes at 893 K, which is attributed to the decomposition of the polymer and ligand portion in NTAamide (C8). The SiO_2 remained stable above 893 K with a content of 57.24%. According to the TG curve of SiO_2-P, the content of the inert copolymer in the SiO_2-P particles obtained is 11.07 %. By calculating, the content of NTAamide(C8) in the adsorbent is 35.63 %.

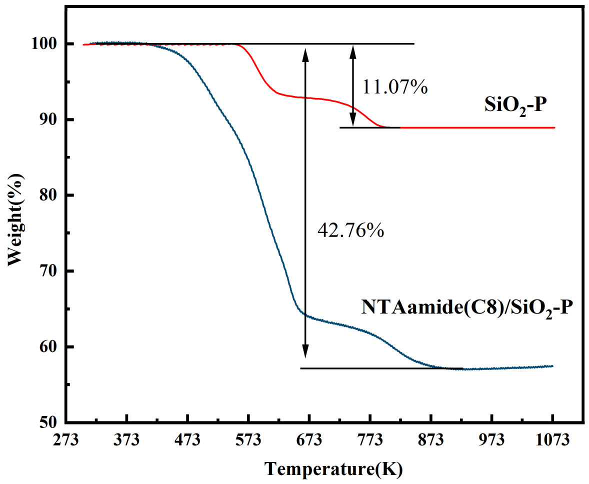

Figure. S2 TG curves of NTAamide(C8)/SiO_2-P and SiO_2-P.

The XRD patterns of NTAamide(C8)/SiO_2-P and Pd-loaded NTAamide(C8)/SiO_2-P (Figure S3) exhibit a wide diffraction peak from 15 to 25°, consistent with the pattern of SiO_2-P. It indicates that silica remains in an amorphous state during the synthesis and adsorption processes.

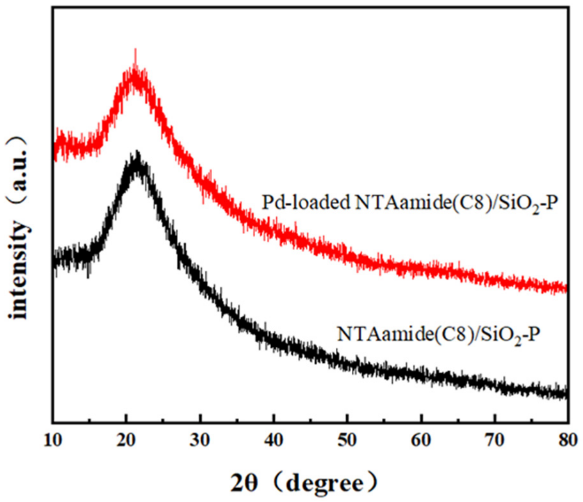

Figure. S3 XRD patterns of NTAamide(C8)/SiO_2-P and Pd-loaded NTAamide(C8)/SiO_2-P.

The N_2-adsorption–desorption isotherm and pore diameter distribution of NTAamide(C8)/SiO_2-P (Figure S4) suggest its mesoporous structure. The calculated specific surface area, pore volume, and average pore size are shown in Table S1.

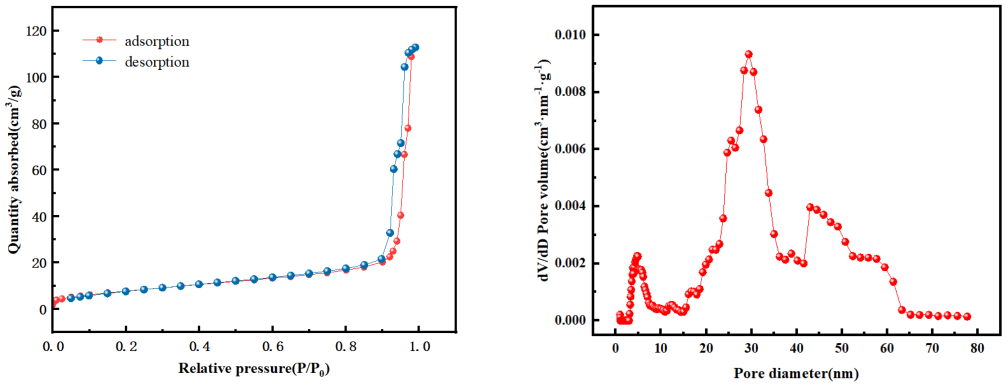

Figure. S4 N_2-adsorption–desorption isotherm and pore diameter distribution of NTAamide(C8)/SiO_2-P.

Table. S1 Main parameters of SiO_2-P and NTAamide(C8)/SiO_2-P obtained from BET.

Materials	BET surface area(m^2/g)	Pore volume (cm^3/g)	Average pore diameter(nm)
NTAamide(C8)/SiO_2-P	26.4	0.17	29.4
